# HAV-Pose: synergizing hybrid attention networks and spherical visibility fields for obstacle-aware fruit harvesting

**DOI:** 10.3389/fpls.2026.1887766

**Published:** 2026-07-13

**Authors:** Yikun Huang, Shuyan Xu, Yulin Zhong, Hao Chen, Hefei Lin, Riqing Chen

**Affiliations:** 1 College of Computer and Information Sciences, Fujian Agriculture and Forestry University, Fuzhou, Fujian, China; 2 Concord University College, Fujian Normal University, Fuzhou, China; 3 Minnan University of Science and Technology, Quanzhou, Fujian, China; 4 Fuzhou Vegetable Science Research Institute, Fuzhou, Fujian, China; 5 Fujian Key Lab of Agricultural Internet of Things Applications, Sanming University, Sanming, Fujian, China

**Keywords:** agricultural robotics, crown pepper harvesting, HAV-Pose, obstacle-aware grasping, Spherical Voxel-based Visibility Field

## Abstract

The timely removal of crown peppers is a critical agronomic procedure to balance vegetative and reproductive growth. However, automating this task is challenged by severe green-on-green occlusion in dense branching structures and high collision risks within narrow Y-shaped stems. Existing robotic systems suffer from feature erosion when detecting camouflaged targets and lack geometric awareness for safe grasping. To address these challenges, this study proposes Hybrid Attention and Visibility-field based Pose estimation (HAV-Pose), a cascaded perception–planning framework. First, an enhanced detection network based on YOLO11 integrates the original, plug-and-play Hybrid Attention Weighted Convolution (HAWConv) and the RepC3k module, which fuses C3k2 with re-parameterized convolutions to balance inference speed and feature representation. Second, to bridge perception and execution, we propose a Spherical Voxel-based Visibility Field (SVVF) algorithm that transforms complex 3D obstacle avoidance into an efficient 2D visibility search centered on the picking point. SVVF employs a deterministic Max-Margin Optimization Strategy to directly compute collision-free 6D grasping poses with maximal safety margins at millisecond-level efficiency. Extensive experiments evaluate the performance of HAV-Pose. Through benchmarking against 14 SOTA models, structural ablation studies, attention mechanism comparisons, and pixel-wise Euclidean distance evaluations, HAV-Pose achieves a mAP@50 of 90.0% (+8.2%) with high localization precision. In heavily occluded scenarios, SVVF attains a robustness rate of 65.4%, outperforming stochastic baselines (56.8%) with only 67 ms additional computation. Furthermore, generalization is validated across four datasets (Crown Pepper, Green Pepper, Eggplant, and Strawberry) and confirmed through real-world harvesting trials, demonstrating reliable perception–action coupling.

## 1 Introduction

Chili peppers are among the most economically significant vegetable crops globally, serving as a vital source of vitamins and bioactive compounds. With the rapid advancement of Agriculture 4.0, the modernization of pepper cultivation has become urgent due to the intensifying shortage of agricultural labor and the rising costs of manual operations. In facility horticulture, harvesting is the most labor-intensive operation, with harvesting labor accounting for approximately 60–70% of the entire production process (Zhang et al., 2024). Consequently, the development of intelligent harvesting robots to replace manual labor has become a research hotspot in agricultural engineering.

While robotic harvesting of mature fruits has seen progress, a critical agronomic procedure remains largely unaddressed: the removal of the Crown Pepper (scientifically defined as the fruit developing at the first branching node of the main stem).From a physiological perspective, the Crown Pepper acts as a dominant “nutrient sink,” competing aggressively for assimilates and inhibiting the vegetative growth of the upper canopy. Therefore, its timely removal is essential for ensuring high yield and quality in subsequent fruiting stages. However, automating this task is far more challenging than harvesting general canopy fruits. The Crown Pepper is situated in the narrow “Y-shaped” bifurcation, resulting in severe “green-on-green” occlusion by dense stems and leaves. Furthermore, the robotic end-effector faces a high risk of collision with the main stem when navigating this constrained space, demanding a perception system that goes beyond simple detection.

To address the challenges of agricultural visual perception, early research primarily relied on traditional machine learning techniques that incorporated color space transformations and handcrafted feature extraction (Gill et al., 2023). However, these approaches often lacked robustness when confronted with illumination variations and complex unstructured backgrounds. With the advent of deep learning, Convolutional Neural Networks (CNNs) have emerged as the de facto standard. Two-stage detectors, represented by Faster R-CNN and Mask R-CNN, have demonstrated high precision in fruit detection tasks (Wang et al., 2022b; Siricharoen et al., 2023). Despite their satisfactory accuracy, the high computational latency associated with these models often fails to meet the stringent real-time requirements of robotic harvesting. Consequently, single-stage detectors, particularly the YOLO (You Only Look Once) series, have become prevalent due to their favorable trade-off between inference speed and detection accuracy (Li et al., 2022; Varghese and M., 2024; Wang et al., 2025a; Tian et al., 2025).

Recently, a wave of domain-specific YOLO variants has emerged to address agricultural challenges. For general architectural improvements, Mamba-YOLO (Wang et al., 2024) and Star-YOLO (Lu et al., 2025) introduced State Space Models (SSMs) and lightweight star-shaped operations to enhance detection efficiency. In the specific context of pepper cultivation, researchers have developed targeted models: YOLO-ALW (Wang et al., 2025b) and Chilli-YOLO (Ma et al., 2024) optimized feature extraction for maturity and biological characteristic detection, while MSPB-YOLO (Zheng et al., 2025) focused on multi-site disease identification. Notably, progress has also been made in harvesting-oriented tasks; Pepper-YOLO (Huang et al., 2024) and the attention-driven MDAD-YOLO (Huang et al., 2026) have improved the localization of picking points in complex environments. Nevertheless, the majority of existing studies have focused on detecting salient fruit bodies rather than the tiny and highly camouflaged fruit stems. When standard YOLO architectures are applied to stem detection, the “Feature Erosion” caused by continuous downsampling operations often leads to severe missed detection issues (Xiao et al., 2023).

To mitigate the inherent limitations of standard convolutions in feature extraction, recent research has attempted to integrate attention mechanisms into detection networks to enhance the focus on regions of interest (RoI). Modules such as SE-Net (Hu et al., 2018), CBAM (Woo et al., 2018), and ECA (Wang et al., 2020) have been introduced into agricultural object detection to suppress background noise. For instance, previous studies have improved apple detection performance by embedding CBAM into the YOLO network (Wu et al., 2024). However, these generic attention mechanisms are typically designed for natural images and may not constitute the optimal solution for the unique “green-on-green” texture indistinguishability associated with fruit stems. Furthermore, the vast majority of existing attention modules employ static weights, failing to dynamically adapt to the severe illumination variations and shadows inherent in unstructured field environments. Consequently, there remains a lack of specialized feature enhancement methods capable of simultaneously addressing the scale variations of small targets and the dynamic complexity of field illumination.

Beyond visual detection, bridging the gap between perception and execution remains a critical bottleneck. Accurate detection alone guarantees neither successful harvesting nor safe interaction; the robot must simultaneously determine a collision-free grasping pose. Existing harvesting robotic systems typically rely on simplified geometric assumptions, such as computing the centroid of bounding boxes or utilizing surface normal estimation (Arad et al., 2020). While computationally efficient, these local methods often overlook the global distribution of surrounding obstacles—such as rigid branches or growth lines—resulting in high collision rates. Conversely, traditional motion planning algorithms, such as Rapidly exploring Random Trees (RRT) or Probabilistic Roadmaps (PRM), can generate safe paths but require the construction of complex global maps and suffer from high computational latency, rendering them unsuitable for continuous, high-speed harvesting cycles (Wang et al., 2022a). Additionally, some researchers have achieved optimal harvesting viewpoints by randomly changing perspectives multiple times and calculating information gain using a scoring function. Although this approach can address occlusion issues and improve fruit harvesting success rates, it requires significant time cost (Burusa et al., 2024; Zaenker et al., 2023). Consequently, it is imperative to develop a holistic system that integrates fine-grained detection with lightweight, geometry-aware planning strategies.

To address the aforementioned challenges, this study proposes a robotic harvesting framework named HAV-Pose. Based on the YOLO11 architecture, this enhanced detection network is specifically customized for the precise localization of fruit stems in unstructured environments. First, to overcome texture ambiguity and feature erosion, we design a lightweight Hybrid Attention Weighted Convolution (HAWConv) module. By dynamically recalibrating features across both channel and spatial dimensions, HAWConv effectively suppresses background noise from dense foliage. Second, to ensure real-time performance on edge devices, we introduce the RepC3k module. This unit reconstructs the backbone by fusing Reparameterized Multi-Branch Convolutions (RepMBConv) (Wang et al., 2025c) into the C3k2 block, achieving a favorable trade-off between inference speed and feature extraction capability. Finally, to bridge the gap between perception and execution, we propose the Spherical Voxel-based Visibility Field (SVVF) algorithm. By transforming the complex 3D obstacle avoidance problem into a 2D visibility map search, SVVF achieves sub-second level resolution of collision-free 6D picking poses.

The main contributions of this study are summarized as follows:

- Proposes HAWConv, a novel attention-augmented plug-and-play operator designed to seamlessly replace standard convolutions.
- Introduces the SVVF algorithm, which transforms 3D obstacle avoidance into a 2D visibility search, facilitating the sub-second level generation of collision-free approach vectors.
- Constructs a dataset with hierarchical semantic annotations, providing a rigorous benchmark for joint object detection and pose estimation in highly occluded environments.
- Conducts extensive benchmarks against 15 SOTA models across four crop domains, demonstrating minimal lateral localization deviation and strong cross-domain generalization.

## 2 Experimental materials

### 2.1 Data acquisition

To rigorously validate the robustness of the perception system in unstructured agricultural environments and assess its cross-crop generalization capabilities, we constructed a multidimensional experimental database comprising four crops: Crown Pepper, Green Pepper, Eggplant, and Strawberry. This dataset encompasses diverse geographical locations, data acquisition devices, and complex lighting conditions.

**Figure 1:**
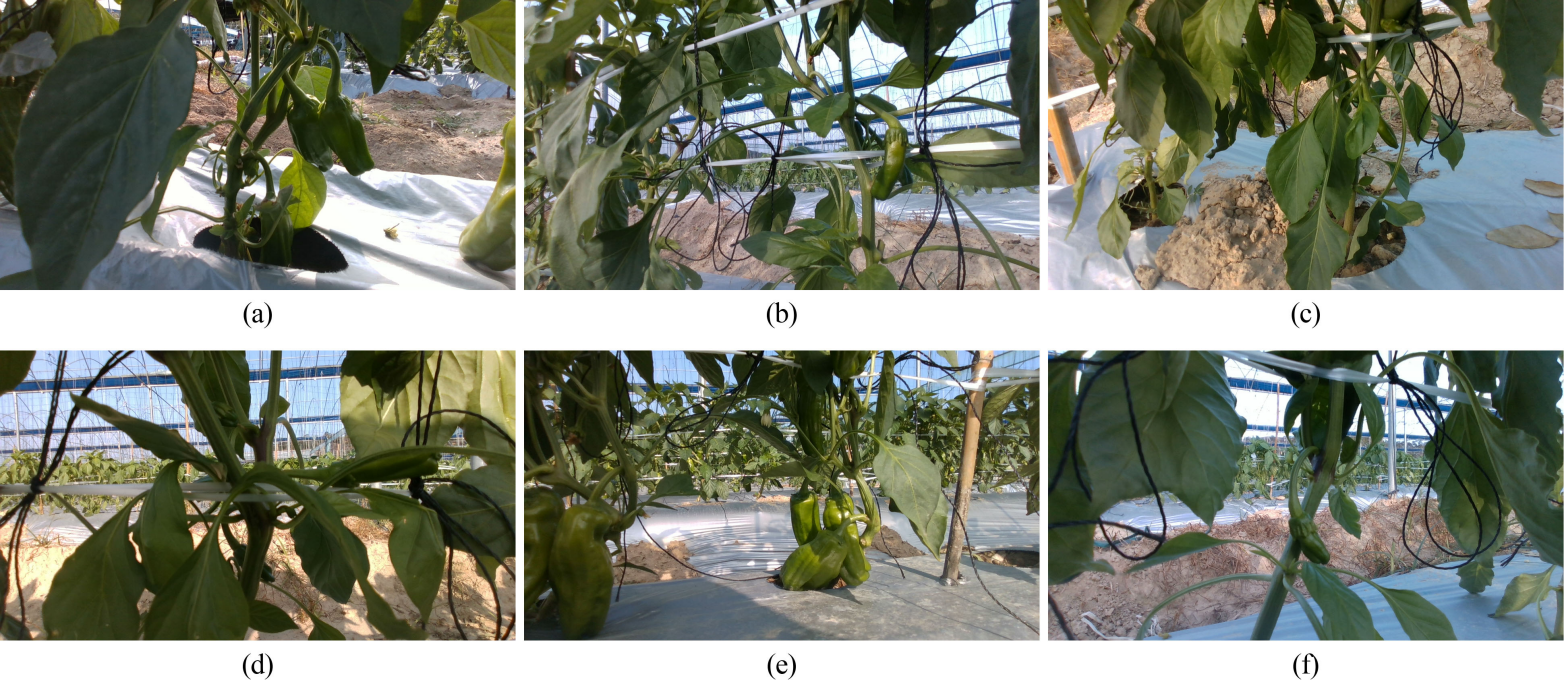
Representative samples from the Crown Pepper dataset illustrating diverse field conditions encountered during data collection. **(a)** Backlighting with strong shadow contrast. **(b)** Front lighting with a bright background. **(c)** Side lighting with soil background clutter. **(d)** Complex Obstacles: the target peduncle is surrounded by dense foliage on multiple sides under backlighting. **(e)** Stem Side Occlusion: the target is partially occluded by adjacent hanging fruits and stems. **(f)** No Occlusion: the target peduncle is clearly visible with minimal surrounding obstacles.

The primary dataset focuses on Crown Pepper, collected on November 3, 2024, at a planting base in Zhangpu County, Fujian, China (24^°^06^′^30.1^′′^*N*,117^°^43^′^55.3^′′^*E*). Images were acquired using an Intel RealSense D435i RGB-D camera at a resolution of 1280×640. To capture the realistic diversity of field environments, data collection spanned from morning (08:00) to dusk (17:00), covering various natural lighting conditions such as front lighting, backlighting, side lighting, and strong shadows. The camera was positioned at a distance of 0.3m to 1.0m from the targets, capturing images from multiple viewpoints (eye-level, upward, and downward) to simulate the complex visual inputs encountered by robotic arms. A total of 1,246 images were collected and randomly divided into training, validation, and testing sets in a 7:1.5:1.5 ratio. As shown in **Figures 1a–c**, this dataset is characterized by severe foliage occlusion, fruit overlapping, and background clutter under diverse lighting conditions. Furthermore, **Figures 1d-f** illustrates the three occlusion categories used to stratify the SVVF evaluation test set: Complex Obstacles, Stem Side Occlusion, and No Occlusion, providing a solid foundation for both model training and systematic obstacle-aware planning evaluation.

To further verify the system’s universality, three additional datasets were introduced. First, the Green Pepper dataset (Huang et al., 2024) was collected on May 2, 2024, in Fuqing, Fujian, China, using the same RealSense D435i camera (1280 × 720), comprising 1,434 images with 2,381 fruit instances. Second, the Eggplant dataset (Huang et al., 2026) was collected on December 12, 2024, at the Institute of Scientific Vegetables, Fuzhou, China (25^°^55^′^23^′′^N,119^°^14^′^41^′′^E). Captured using an Oppo Find X7 smartphone, this dataset consists of 1,451 images and specifically targets lighting robustness, including 670 bright, 111 low-light, and 670 night-time scenes, totaling 2,727 annotated instances.

Finally, the Strawberry dataset was sourced from the StrawDI benchmark, collected by Pérez-Borrero et al. (2020) in the province of Huelva, Spain. This dataset comprises 3,100 images, partitioned into training (2,800), validation (100), and testing (200) subsets. To ensure consistency with the annotation standards applied to the previous three datasets, we re-annotated the entire dataset following the specific implementation protocols of this study.

To provide a comprehensive characterization of the experimental data, **Table 1** presents the statistical summary of all four datasets, including class distribution and instance counts across training, validation, and testing splits. The Crown Pepper dataset contains three hierarchical annotation categories that co-occur in every image, yielding a near-balanced instance ratio of approximately 1.87:1.38:1.00. The Green Pepper dataset exhibits a higher average instance density of approximately 3.8 instances per image, reflecting the typical clustering growth pattern of peppers. The Strawberry dataset contains two annotation categories (Ripe and Unripe) sharing the same image set, with a training-split class imbalance ratio of approximately 5.3:1; no resampling or class weighting was applied, as YOLO-based detectors demonstrate robustness to moderate imbalance through focal loss optimization.

**Table 1:** Statistical summary of the four experimental datasets, including class distribution across training, validation, and testing splits.

Dataset	Class	Train (img)	Val (img)	Test (img)	Train (inst.)	Val (inst.)	Test (inst.)
Crown Pepper	Crown Pepper	872	186	188	960	229	211
	First Branching	872	186	188	712	167	159
	Planting Hole	872	186	188	514	122	111
Green Pepper	Pepper	1261	86	87	4,797	328	410
Eggplant	Eggplant	1015	217	219	1912	414	401
Strawberry	Ripe	2800	100	200	13,648	460	980
	unRipe	2800	100	200	2,586	112	152

### 2.2 Dataset annotation

**Figure 2:**
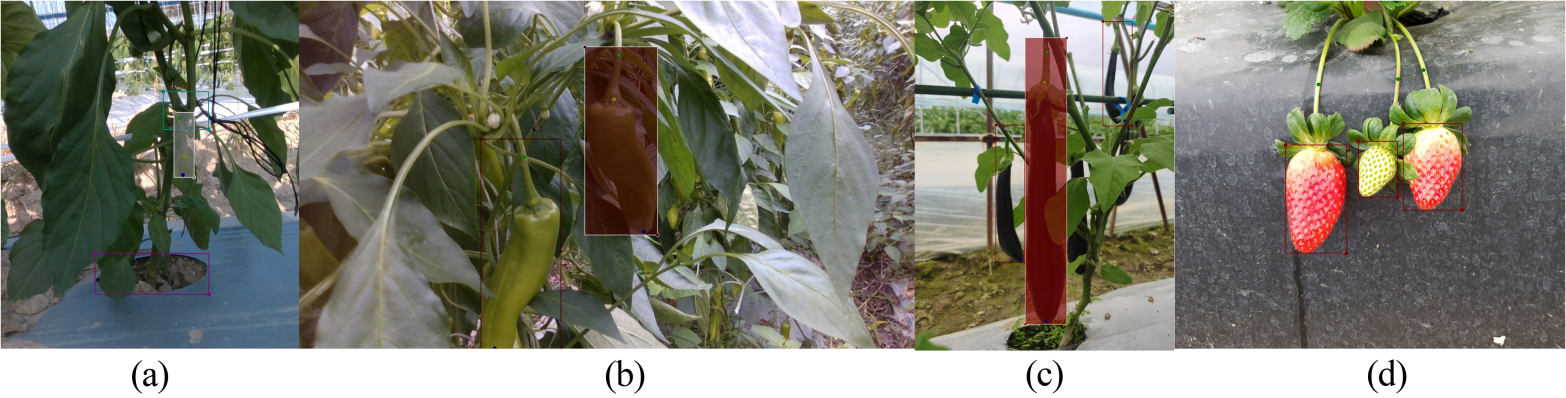
Representative samples from the four experimental datasets: **(a)** Crown Pepper, **(b)** Chili Pepper, **(c)** Eggplant, and **(d)** Strawberry. Annotation Protocol: All datasets feature bounding boxes for target detection. Uniquely, **(a)** includes environmental annotations for the Planting Hole and first branching. regarding fruit pose, **(a-c)** utilize three keypoints (picking point, fruit top, and fruit bottom), whereas **(d)** employs a single keypoint on the peduncle to indicate the picking location.

All datasets were annotated using the Labelme tool. For Crown Pepper, a hierarchical annotation scheme was adopted: bounding boxes were labeled for the Crown Pepper, First Branching Node, and Planting Hole; for pose estimation, three keypoints were marked: Picking point, Fruit Base (Top), and Fruit Tip (Bottom). The Green Pepper and Eggplant datasets were annotated with a single bounding box for the fruit and the three corresponding keypoints. For the Strawberry dataset, we re-annotated the bounding boxes and a single Picking point according to the protocols of this study. Representative annotated samples are shown in **Figure 2**.

## 3 Methodology

### 3.1 Proposed system overview

**Figure 3:**
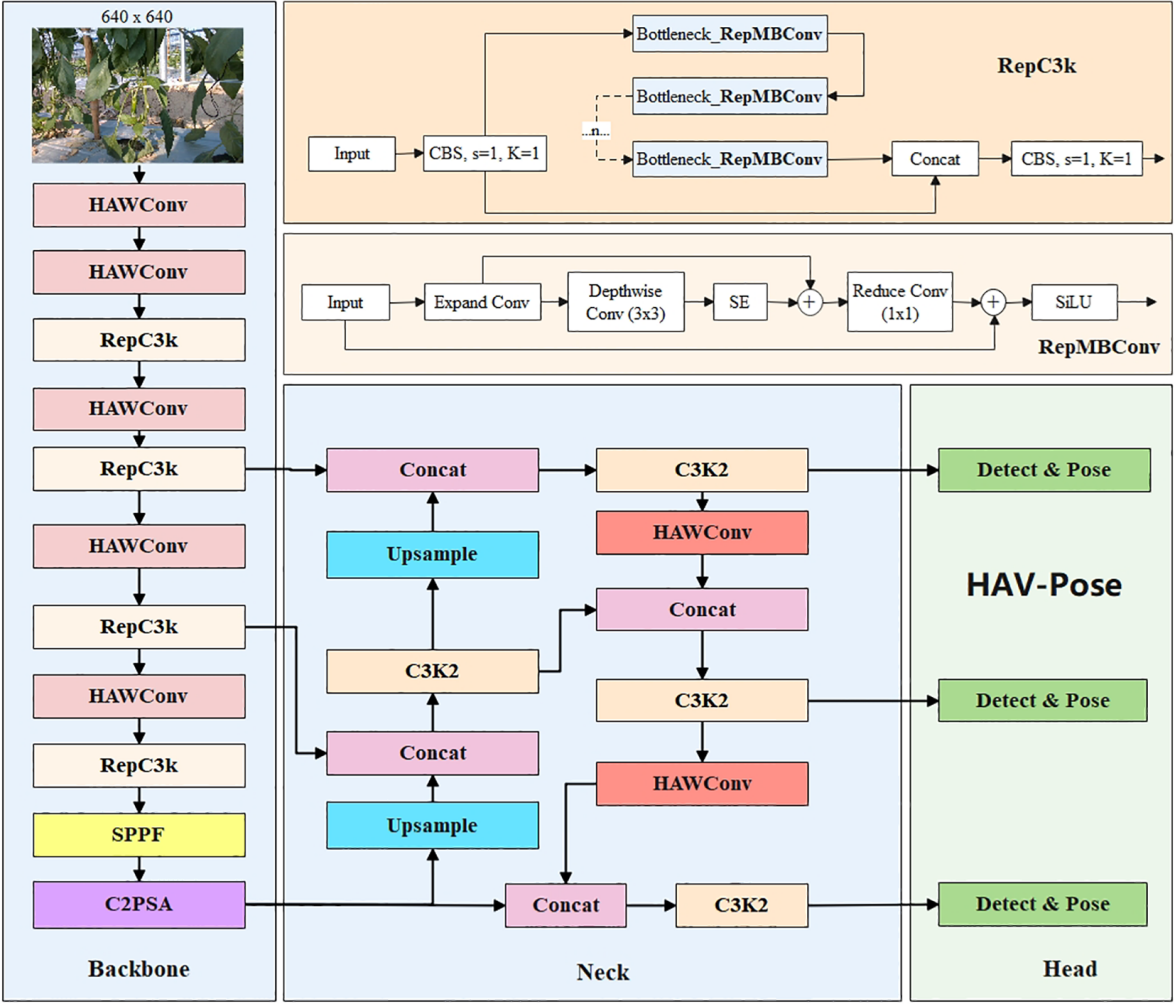
The overall architecture of the proposed HAV-Pose network. The network integrates the HAWConv operator (structure detailed in Section 3.2) and lightweight backbone components. The top-right panel details the internal structure of the RepC3k module and its core unit, RepMBConv.

**Figure 4:**
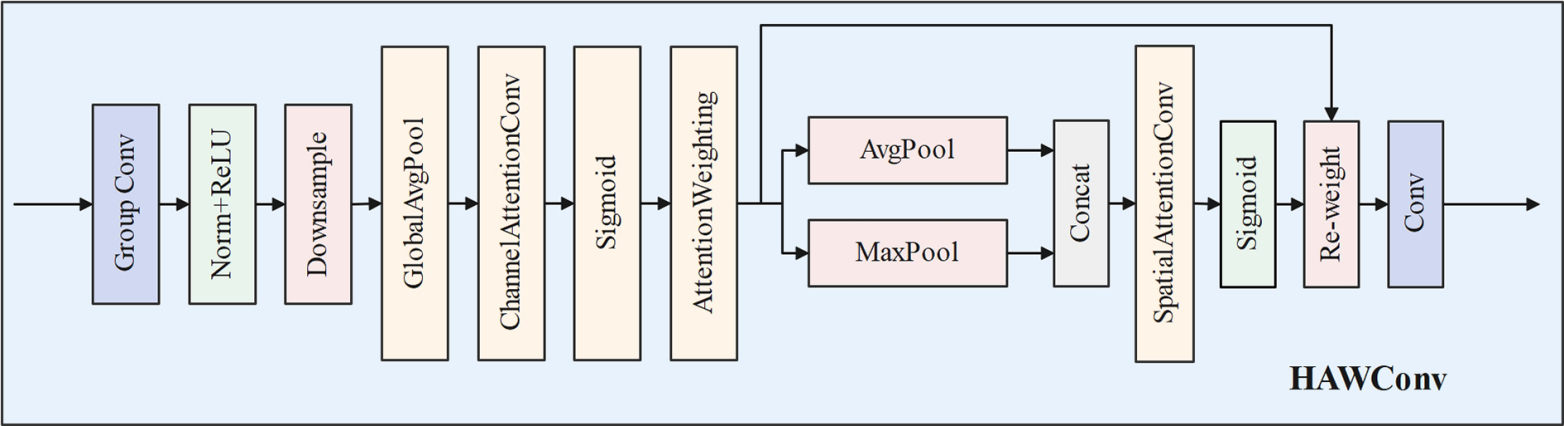
Architecture of the HAWConv.

**Figure 5:**
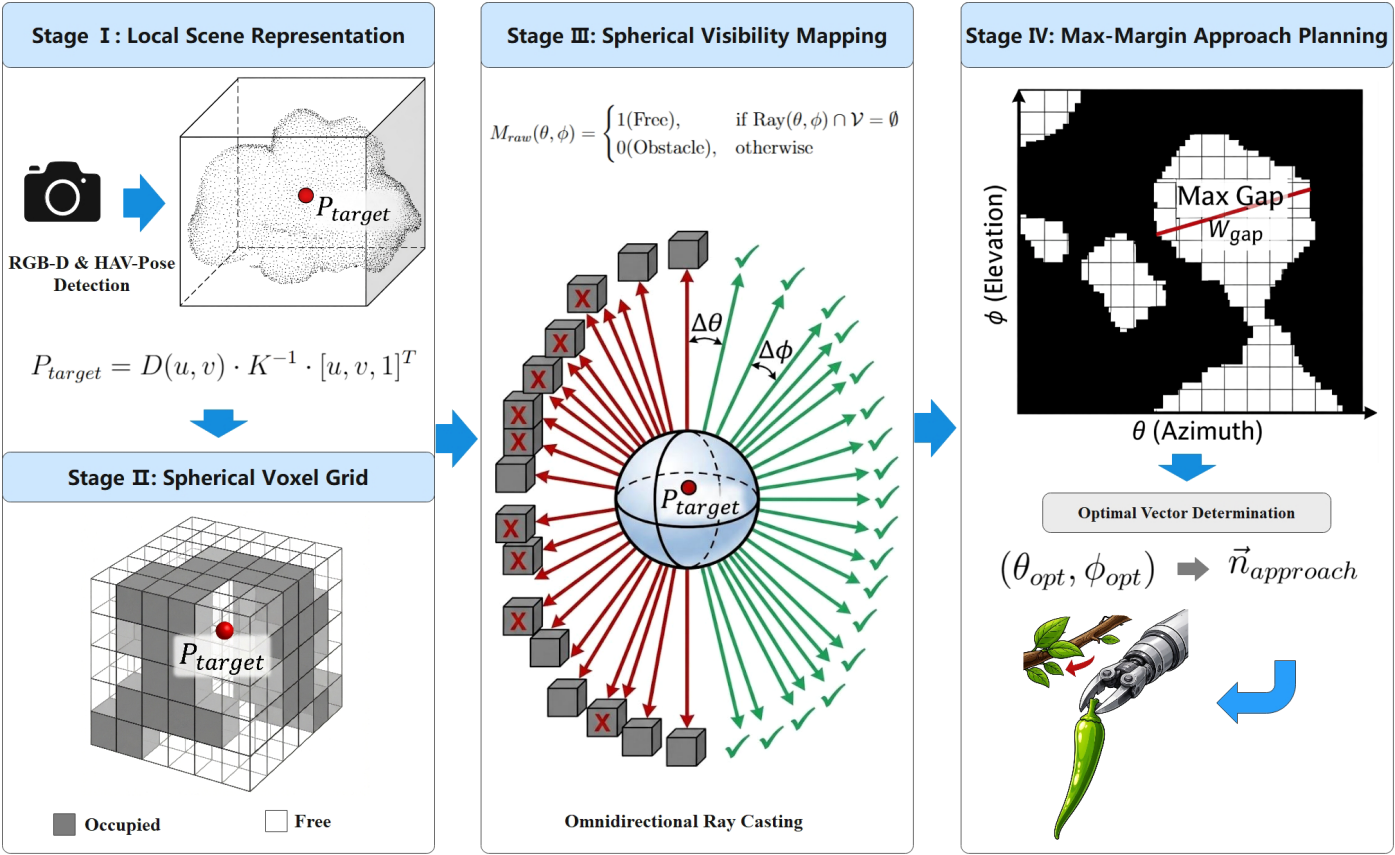
The schematic pipeline of the SVVF algorithm. The process consists of four sequential stages: (I) 3D Scene Reconstruction and Target Localization, (II) Local Spherical Voxelization, (III) Spherical Visibility Mapping, and (IV) Max-Margin Approach Planning.

To address the challenges of “green-on-green” occlusion and ensure safe robotic intervention in unstructured agricultural environments, we propose a cascaded perception-planning framework. The proposed framework integrates the visual network **Figure 3** (Network Architecture), which embeds our HAWConv block (**Figure 4**) as a key convolutional structure, with the robotic planning pipeline **Figure 5** (System Pipeline). As illustrated in this combined architecture, the system seamlessly couples high-precision visual detection with geometric obstacle avoidance. The workflow proceeds through two distinct phases: Visual Perception via HAV-Pose and Geometric Planning via SVVF.

The pipeline initiates with a 640 × 640 RGB input fed into the HAV-Pose network. To enable real-time deployment on edge devices without compromising feature extraction capability, the network backbone is constructed using the RepC3k block (detailed in Section 3.3). This module leverages structural re-parameterization to decouple training complexity from inference latency, achieving high computational efficiency (low GFLOPs). Furthermore, to mitigate background noise from dense foliage, we embed the HAWConv operator (detailed in Section 3.2), which employs hybrid attention mechanisms to highlight slender stem features. The network concludes with a decoupled head that simultaneously predicts the bounding box, object confidence, and three semantic keypoints: the picking point (*P_pick_*), fruit base (*P_top_*), and fruit tip (*P_bottom_*).

Following visual perception, the detected 2D keypoints initiate the geometric planning phase. Specifically, the system projects the 2D picking point (*P_pick_*) into 3D space by retrieving the corresponding depth value, thereby establishing the precise spatial location of the target stem. This 3D picking point serves as the spherical center for the Spherical Voxel-based Visibility Field (SVVF) algorithm (detailed in Section 3.4). In this stage, the unstructured environment surrounding the stem is discretized into a spherical voxel grid, effectively transforming the complex 3D obstacle avoidance problem into an efficient 2D visibility search task. Ultimately, the algorithm computes an optimal collision-free approach vector ${\vec{n}}_{\text{approach}}$ that maximizes the safety margin for the robotic gripper.

### 3.2 HAWConv: hybrid attention weighted convolution

Standard convolutions treat spatial and channel dimensions equally, often failing to distinguish slender pepper stems from complex background textures. To address this, we propose HAWConv, a lightweight operator that integrates a hybrid attention mechanism to enhance target saliency, as illustrated in **Figure 4**. The module first employs Group Convolution and downsampling to generate a compact feature representation, **F***_in_*, reducing parameter redundancy. To explicitly model the inter-channel dependencies (“what to pay attention to”), we employ a channel attention mechanism. Global Average Pooling (GAP) aggregates spatial contexts, followed by a convolution layer to generate channel weights. The channel-refined feature **F**^′^ is computed as shown in Equation 1:

(1) $$F^{\prime }=Mc⊗Fin=\sigma \left(Wc\left(\text{GAP}\left(Fin\right)\right)\right)⊗F_{in}$$

where ⊗ denotes element-wise multiplication, *σ* is the Sigmoid function, and W*_c_* represents the channel interaction convolution. Subsequently, the spatial attention mechanism addresses the “where to focus” aspect. A dual-pooling strategy (Average and Max pooling) captures both dominant features and background context. Through convolution and Sigmoid activation, the generated spatial weight map accurately localizes the stem contours. Finally, the features are calibrated via a Re-weight operation and processed by a standard convolution layer to yield the final output **Y**, as defined in Equation 2:

(2) $$Y=Wout\left(\sigma \left(Ws\left(\left[\text{Avg}\left(F^{\prime }\right);\text{Max}\left(F^{\prime }\right)\right]\right)\right)⊗F^{\prime }\right)$$

where [·;·] denotes concatenation, and W*_s_*represents the spatial attention convolution. This hybrid mechanism effectively suppresses background noise and significantly enhances the feature representation of small-scale stems.

While HAWConv adopts a sequential channel-then-spatial attention paradigm that may appear superficially similar to CBAM (Woo et al., 2018), it differs in three structural aspects that are particularly relevant to “green-on-green” peduncle detection. First, CBAM’s channel branch employs a shared MLP with dimensionality reduction ratio *r* (typically *r* = 16), which discards fine-grained inter-channel relationships critical for discriminating green stems from visually similar foliage. HAWConv replaces this bottleneck with a full-rank depthwise convolution W*_c_*applied directly on the GAP-aggregated descriptor, preserving the complete *C*-dimensional channel representation. Second, CBAM computes its spatial attention mask from the raw input feature, whereas HAWConv’s spatial attention explicitly operates on the channel-refined feature **F**^′^, ensuring that spatial localization is guided by foliage-suppressed representations—a distinction that matters when the target and background share nearly identical low-level texture statistics.

Third, CBAM functions as an auxiliary module appended after an unchanged standard convolution, whereas HAWConv integrates feature compression, attention recalibration, and output projection into a unified operator that directly replaces standard convolutions in the backbone. These structural differences collectively address the feature erosion problem inherent in standard convolutions when applied to camouflaged small-scale targets in unstructured agricultural environments.

### 3.3 RepC3k: re-parameterized feature aggregation for efficient inference

In the specific context of robotic pepper harvesting, the high visual ambiguity between green stems, fruits, and foliage poses a significant challenge for feature extraction. Standard aggregation modules in YOLOv11 (Khanam and Hussain, 2024), such as C3k2, rely on fixed bottleneck structures; enhancing their capacity to discriminate subtle stem textures often requires increasing network depth, which inevitably imposes high latency on edge computing devices. To reconcile the conflict between representational capacity and inference efficiency, we propose the RepC3k module. This architecture inherits the efficient CrossStage Partial (CSP) topology of the original C3k2 but innovatively integrates Structural Reparameterization to redesign the internal processing units. By replacing standard bottlenecks with RepMBConv blocks, the module decouples the training-time architecture from the inference-time structure, ensuring that the network can learn complex “green-on-green” features without incurring computational penalties during deployment.

Specifically, the RepC3k module leverages a dual-mode strategy. During the training phase, each RepMBConv unit utilizes a multi-branch topology comprising a 3×3 depthwise convolution, a 1 × 1 expansion convolution, and parallel identity or over-parameterized branches. This complex connectivity enriches the gradient flow space, allowing the model to capture diverse high-frequency spatial details—such as the sharp edges of stems and the vein textures of leaves—which are critical for target differentiation. For inference, we exploit the linearity of convolution operations to mathematically fuse this multi-branch structure into a single equivalent 3 × 3 kernel. Let *W_eq_*and *b_eq_*denote the re-parameterized weights and biases; the forward pass is simplified to *F_out_*= *F_in_*∗ *W_eq_*+ *b_eq_*. This transformation effectively eliminates the memory access cost (MAC) associated with fragmented branches, thereby collapsing the high-capacity multi-path network into a compact, single-path stream that meets the real-time requirements of robotic harvesting.

### 3.4 Obstacle-aware pose estimation via Spherical Voxel-based Visibility Field

To bridge the gap between 2D visual perception and 3D robotic execution, we propose the Spherical Voxel-based Visibility Field (SVVF) algorithm. This global planning method transforms the complex 3D obstacle avoidance problem into an efficient 2D visibility search task. As illustrated in **Figure 5**, the algorithm orchestrates collision-free grasping through four sequential stages:

#### 3.4.1 3D scene reconstruction and preprocessing

The prerequisite for safe planning is an accurate geometric representation of the workspace. We first register the RGB image with the depth map *D*. Based on the pinhole camera model, the 2D visual data is back-projected into 3D space. For an arbitrary pixel (*u,v*), its 3D coordinate $P\in {\mathbb{R}}^{3}$ is computed by retrieving the corresponding depth value *D*(*u,v*):

(3) $$P=D\left(u,v\right)\cdot K^{-1}\cdot {\left[u,v,1\right]}^{T}$$

where *K* denotes the intrinsic matrix. To mitigate noise caused by leaf diffraction and specular reflections, we apply Statistical Outlier Removal (SOR) and Voxel Grid Downsampling to generate a refined point cloud. Simultaneously, the specific 3D centroid of the target stem, denoted as *P_target_*, is precisely reconstructed by applying Equation 3 to the detection center (*u_c_,v_c_*).

#### 3.4.2 Local spherical voxelization

Using the refined point cloud and target centroid, we model the local environment. An ROI centered at *P_target_* is cropped to define the potential collision space. This continuous space is then discretized into a voxel grid V with resolution *v_res_*. Voxels containing points from the preprocessed cloud are marked as “Occupied” to represent obstacles (e.g., branches and foliage), while empty voxels are marked as “Free”.

#### 3.4.3 Spherical visibility mapping

To efficiently map obstacle distribution, we establish a local spherical coordinate system at *P_target_*and perform Omnidirectional Ray Casting. Rays *R*(*θ,ϕ*) are emitted at discrete intervals to check for collisions against V. We first define a raw binary visibility map *M_raw_*, as defined in Equation 4:

(4) $$M_{raw}\left(\theta ,\phi \right)=\{\begin{array}{ll} 1\left(\text{Free}\right), & \text{if Ray}\left(\theta ,\phi \right)\cap V=\emptyset \\ 0\left(\text{Obstacle}\right), & \text{otherwise} \end{array}$$

To account for the gripper’s physical volume, morphological dilation is applied to the obstacle regions in *M_raw_*. This generates the final Spherical Visibility Map (SVM), denoted as *M*, ensuring a sufficient safety buffer for insertion.

#### 3.4.4 Max-margin approach planning

Optimal pose determination balances geometric safety margins with kinematic constraints. We employ a row-wise scanning strategy on *M* to identify the set of continuous candidate gaps $C=\left\{C_{1},\ldots ,C_{N}\right\}$, where each *C_i_* is parameterized by its width *W_i_*and center $\left(\theta_{i},\phi_{i}\right)$. These candidates are evaluated using a multi-objective scoring function *S*(*C_i_*), as defined in Equation 5:

(5) $$S\left(C_{i}\right)=W_{i}\cdot \text{exp }\left(-\frac{\left|\phi_{i}-\phi_{pref}\right|}{\sigma_{\phi }}\right)\cdot \text{exp }\left(-\frac{d\left(\theta_{i},\theta_{arm}\right)}{\sigma_{\theta }}\right)$$

Here, *W_i_* is the base gap width. *ϕ_pref_* (set to *π/*2) and *θ_arm_* represent preferred configurations for horizontal entry and manipulator base alignment, respectively. *d*(·,·) computes the shortest geodesic distance on *S*^1^, while σ*_ϕ_* and σ*_θ_* regulate penalty sensitivity analogously to Gaussian standard deviations.

Ultimately, the algorithm selects the candidate *C_opt_* maximizing *S*(*C_i_*). Its geometric center (*θ_opt_*, *ϕ_opt_*) is transformed into a Cartesian unit vector ${\vec{n}}_{approach}\in {\mathbb{R}}^{3}$, serving as the final approach instruction.

## 4 Experiments

### 4.1 Implementation details

All experiments were performed on a high-performance workstation powered by an AMD EPYC 7402 CPU and an NVIDIA GeForce RTX 4090 GPU (24GB VRAM) with 60GB of system memory. The software stack consisted of Ubuntu 22.04, the PyTorch 2.2 deep learning framework, and the CUDA 12.1 acceleration library. During training, input images were resized to a resolution of 640 × 640. We employed the Stochastic Gradient Descent (SGD) optimizer. The hyperparameters were configured as follows: an initial learning rate of 0.01, momentum of 0.937, and weight decay of 0.0005. The batch size was set to 16, and the models were trained for a total of 300 epochs.

### 4.2 Evaluation metrics

For object detection and picking point localization tasks, we utilize standard Precision (P) and Recall (R) metrics. To evaluate the overall performance, we calculate the Mean Average Precision (mAP) as defined in Equation 6.

(6) $$mAP=\frac{1}{C}\sum_{i=1}^{C}\int_{0}^{1}P\left(R\right)dR$$

To rigorously quantify the regression accuracy of the picking point (*P_pick_*), we utilize pixel-level error metrics. We calculate the Mean Euclidean Distance Error (MEDE), which measures the average spatial deviation between the predicted coordinates $\left({\hat{x}}_{i},{\hat{y}}_{i}\right)$ and the ground truth (*x_i_*, *y_i_*), as defined in Equation 7. Additionally, the Standard Deviation (SD) is computed to assess the stability of the regression, as shown in Equation 8.

(7) $$MEDE=\frac{1}{N}\sum_{i=1}^{N}\sqrt{{\left(x_{i}-{\hat{x}}_{i}\right)}^{2}+{\left(y_{i}-{\hat{y}}_{i}\right)}^{2}}$$

(8) $$SD=\sqrt{\frac{1}{N}\sum_{i=1}^{N}{\left(d_{i}-\mu \right)}^{2}}$$

where *N* is the number of samples, *d_i_*represents the Euclidean error for the *i*-th sample, and *µ* is the mean error.

To assess the feasibility of deploying the model on resource-constrained edge devices, we report three hardware-related indicators: Parameters (Params), representing the model size; GFLOPs, measuring the computational complexity in billions of floating-point operations; and Frames Per Second (FPS), indicating the real-time inference speed on the target hardware.

The effectiveness of the SVVF algorithm is evaluated using Average Planning Time (*T_plan_*) to measure latency. Additionally, we employ Success Rate (SR) to indicate the proportion of trials generating feasible paths, as calculated in Equation 9. Furthermore, the Collision Rate (CR) is used to quantify the safety level by identifying trajectories that violate safety margins, as defined in Equation 10.

(9) $$SR=\frac{N_{success}}{N_{total}}\times 100\%$$

(10) $$CR=\frac{N_{collision}}{N_{total}}\times 100\%$$

where *N_total_* is the total number of test cases, *N_success_* denotes trials generating valid paths satisfying kinematic constraints, and *N_collision_* represents trials resulting in obstacle collisions.

### 4.3 Comparative analysis with state-of-the-arts

We evaluated the HAV-Pose model against 14 state-of-the-art (SOTA) methods. The comparison scope encompasses standard YOLO variants ranging from YOLOv5n-pose (Jocher et al., 2020) to YOLOv13n-pose (Lei et al., 2025), advanced detection architectures such as Star-YOLO and Mamba-YOLO, and domain-specific models optimized for agricultural scenarios, including Pepper-YOLO, Chilli-YOLO, MSPB-YOLO, and YOLO-ALW. Detailed quantitative results are summarized in **Table 2**. Notably, among the standard YOLO series (v5 to v13), YOLOv11n-pose achieved the highest mAP_50:95_ for crown pepper picking point localization. Consequently, it was selected as the baseline model for this study.

**Table 2:** Performance comparison with state-of-the-art methods on the crown pepper dataset.

Models	Params	GFLOPs	FPS	Object detection (%)				Pose estimation (%)			
	(M)			P	R	mAP_50_	mAP_50:95_	P	R	mAP_50_	mAP_50:95_
YOLOv5n-pose	2.25	6.1	333	73.5	70.8	75.8	43.2	80.6	75.8	81.8	74.1
YOLOv6n-pose	4.18	11.5	333	71.2	68.2	72.8	39.3	75.4	74.4	79.3	68.5
YOLOv8n-pose	2.76	7.1	345	79.1	69.2	77.8	41.9	**87.2**	74.9	85.8	75.4
YOLOv9t-pose	1.78	6.7	263	76.2	68.4	75.6	41.9	84.5	72.2	81.7	73.3
YOLOv10n-pose	2.34	6.8	333	79.9	69.9	78.2	42.6	84.8	74.1	84.3	73.9
YOLOv11n-pose	2.65	6.6	323	82.1	72.5	80.9	43.2	81.0	77.3	85.4	76.7
YOLOv12n-pose	2.58	6.1	357	81.3	68.2	79.0	41.4	82.9	75.8	84.8	75.0
YOLOv13n-pose	2.52	6.4	238	79.4	69.7	77.7	42.2	78.2	76.3	81.9	72.7
YOLO-Start	2.01	5.2	217	79.2	59.6	70.2	36.3	83.1	71.6	81.0	70.3
MSPB-YOLO	2.69	6.8	285	82.7	62.6	78.0	44.3	85.7	71.6	84.5	75.7
Pepper-YOLO	1.61	4.7	370	75.7	64.0	73.6	38.8	76.1	74.9	80.7	69.3
YOLO-ALW	2.92	7.1	250	82.8	71.1	79.4	43.4	86.1	73.9	84.2	76.1
Chilli-YOLO	1.99	5.8	345	74.7	66.8	72.6	37.9	77.9	73.7	81.4	71.5
Mamba-YOLO	5.73	12.6	192	83.2	71.6	80.9	44.2	79.3	80.0	86.5	77.9
**HAV-Pose (Ours)**	2.76	8.5	285	**86.0**	**75.9**	**85.6**	**48.5**	86.4	**84.0**	**90.0**	**82.3**

Bold values indicate the performance of our proposed HAV-Pose model, which achieves the best results in most metrics.

In the detection of crown peppers, HAV-Pose achieves competitive performance across core metrics, outperforming existing SOTA models. Compared with the baseline YOLOv11npose, HAV-Pose improves the keypoint pose estimation accuracy (*Pose*-*mAP*_50_) by 4.6% and achieves a gain of 5.6% on the stricter metric (*Pose*-*mAP*_50:95_). Regarding object detection, *Box*-*mAP*_50_ and *Box*-*mAP*_50:95_ increased by 4.7% and 5.3%, respectively. This evidences that the proposed improvement modules effectively capture the features of irregular fruits. Among domain-specific models optimized for pepper recognition, HAV-Pose maintains consistent advantages. For instance, it outperforms the similarly sized MSPB-YOLO by 6.6% in *Pose*-*mAP*. More notably, even when challenged against the parameter-heavy Mamba-YOLO (5.73M parameters), HAV-Pose surpasses it by 3.5% in *Pose*-*mAP*_50_ and 4.4% in *Pose*-*mAP*_50:95_. Furthermore, HAV-Pose achieves an inference speed of 285 FPS on the experimental hardware, exceeding the real-time requirement (30 FPS) and fully meeting the practical deployment needs of agricultural harvesting robots. In summary, HAV-Pose achieves a favorable balance between accuracy, model complexity, and inference speed.

**Figure 6:**
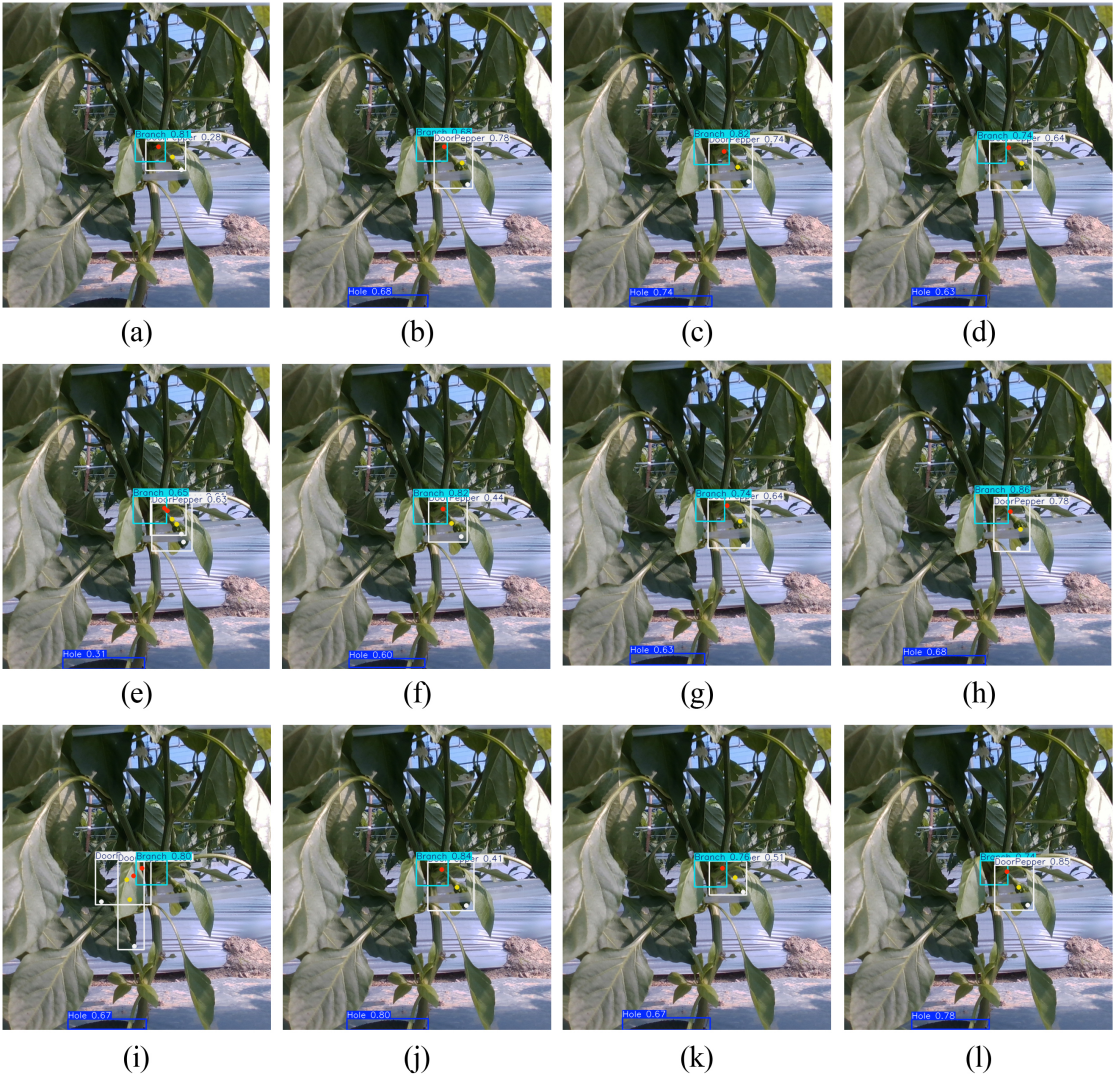
Qualitative comparison of pose estimation results on a representative Crown Pepper sample. The figure displays detection outputs from 12 different models: **(a)**YOLOv5npose, **(b)** YOLOv8n-pose, **(c)** YOLOv9n-pose, **(d)** YOLOv10n-pose, **(e)** YOLOv11n-pose (baseline), **(f)** YOLOv12n-pose, **(g)** YOLOv13n-pose, **(h)** Star-YOLO, **(i)** MSPB-YOLO, **(j)** YOLO-ALW, **(k)** Chilli-YOLO, and **(l)** the proposed HAV-Pose. The grasping pose is indicated by three keypoints: red (picking point), yellow (fruit top), and white (fruit bottom).

To qualitatively illustrate the performance differences among the compared models, **Figure 6** presents a visual comparison across 14 models on a representative test sample. Visual inspection highlights critical baseline limitations: (1) Picking point misalignment in YOLOv5-v13, YOLO-ALW, and Chilli-YOLO, where the red dot drifts off the stem; (2) Pose axis distortion, evidenced by the deviated top/bottom keypoints in YOLOv5-pose and Star-YOLO; (3) False positives in YOLOv9 and MSPB-YOLO due to background clutter; and (4) Redundant detections in the baseline YOLOv11n. In comparison, HAV-Pose substantially reduces these artifacts. It generates unique, tight bounding boxes and aligns all three keypoints precisely with the fruit’s geometry. This visual evidence validates the effectiveness of the integrated HAWConv and RepC3k modules in enhancing feature discriminability and spatial regression accuracy.

### 4.4 Ablation studies

To systematically verify the effectiveness of the proposed modules, we conducted component-wise ablation experiments on the crown pepper dataset using the original YOLOv11n-pose as the baseline. As summarized in **Table 3**, the HAWConv proved to be a critical performance driver. Replacing standard convolutions with HAWConv (Row 3) improved object detection accuracy (*Box*-*mAP*_50_) from 80.9% to 83.4% and pose estimation accuracy (*Pose*-*mAP*_50_) from 85.4% to 88.1%. This improvement indicates that the hybrid attention mechanism within HAWConv effectively re-weights feature maps, allowing the network to focus on the salient texture and geometric features of irregular crown peppers while suppressing background noise.

**Table 3:** Ablation study of proposed modules on the crown pepper dataset.

HAWConv	RepC3k (Backbone)	RepC3k (Neck)	Params (M)	GFLOPs	FPS	Object detection (%)				Pose estimation (%)			
						P	R	mAP_50_	mAP_50:95_	P	R	mAP_50_	mAP_50:95_
×	×	×	2.65	6.6	322	82.1	72.5	80.9	43.2	81.0	77.3	85.4	76.7
×	✓	×	2.75	7.2	294	81.2	68.7	79.9	42.5	81.7	78.3	85.1	75.1
✓	×	×	2.75	8.3	270	82.4	74.4	83.4	46.6	85.7	77.3	88.1	80.5
✓	✓	✓	2.84	8.9	204	84.0	75.4	84.1	46.0	87.4	81.0	88.5	80.5
✓	✓	×	2.76	8.5	285	86.0	75.9	**85.6**	**48.5**	86.4	84.0	**90.0**	**82.3**

Bold values indicate the performance of our proposed HAV-Pose model, which achieves the best results in most metrics.

Regarding the architectural optimization, we observed a strong synergistic effect when combining HAWConv with RepC3k in the backbone. Although RepC3k alone yielded limited gains, its integration with HAWConv (Row 5) achieved the best performance, reaching a peak *Box*-*mAP*_50_ of 85.6% and *Pose*-*mAP*_50_ of 90.0%. This suggests that reparameterized RepC3k blocks efficiently extract semantic representations from the attention refined features provided by HAWConv. Conversely, extending RepC3k to the Neck (Row 4) resulted in performance degradation (*Box*-*mAP*_50_ dropped to 84.1%), implying that the original C3k2 structure remains more suitable for the multi-scale feature fusion stage. Consequently, the configuration in Row 5 was adopted as the final HAV-Pose architecture, achieving competitive accuracy with balanced model complexity (2.76M parameters).

### 4.5 Comparison with attention-enhanced convolutions

To further evaluate the architectural effectiveness of HAWConv, we conducted a comparative analysis against mainstream attention mechanisms, including SE, ECA, CBAM, and Coordinate Attention (CA). To ensure a fair comparison, these conventional attention modules were integrated in a serial “Standard Convolution + Module” configuration. The quantitative results are summarized in **Table 4**.

**Table 4:** Comparative analysis of different attention mechanisms and feature extraction operators.

Model	Params	GFLOPs	FPS	Object detection (%)				Pose estimation (%)			
	(M)			P	R	mAP_50_	mAP_50:95_	P	R	mAP_50_	mAP_50:95_
Baseline	2.65	6.6	323	82.1	72.5	80.9	43.2	81.0	77.3	85.4	76.7
SE-Net	2.68	6.8	263	80.5	71.6	78.9	43.6	81.1	77.1	84.0	75.5
CA	2.68	6.8	256	86.8	67.8	80.4	42.0	83.0	75.4	84.6	75.4
CBAM	2.68	6.9	286	85.5	65.9	80.9	42.5	74.7	82.7	85.7	76.8
ECA-Net	2.67	6.8	256	85.5	75.5	84.1	44.0	88.2	78.0	87.3	79.3
**HAWConv (Ours)**	2.76	8.5	286	86.0	75.9	**85.6**	**48.5**	86.4	84.0	**90.0**	**82.3**

Bold values indicate the performance of our proposed HAV-Pose model, which achieves the best results in most metrics.

As shown in **Table 4**, HAWConv achieves the highest performance across all metrics. Standard attention mechanisms often exhibit limitations: SE and ECA focus primarily on channel dependencies, while CA specifically emphasizes embedding positional information into channel attention. Although CBAM attempts to combine both channel and spatial dimensions, its channel attention branch introduces a dimensionality reduction bottleneck (reduction ratio *r* = 16) that discards fine-grained inter-channel relationships critical for stem-foliage discrimination, and its spatial attention operates on unrefined raw features rather than semantically purified representations. These structural limitations, elaborated in Section 3.2, collectively explain the 4.3% *Pose*-*mAP*_50_ gap between CBAM (85.7%) and HAWConv (90.0%). In contrast, HAWConv leverages a hybrid attention mechanism to simultaneously capture semantic texture (channel) and geometric position (spatial) cues. Consequently, HAWConv outperforms the runner-up ECA-Net by 1.5% in *Box*-*mAP*_50_ (85.6% vs. 84.1%) and 2.7% in *Pose*-*mAP*_50_ (90.0% vs. 87.3%), suggesting its effectiveness in extracting discriminative features from complex backgrounds.

**Figure 7:**
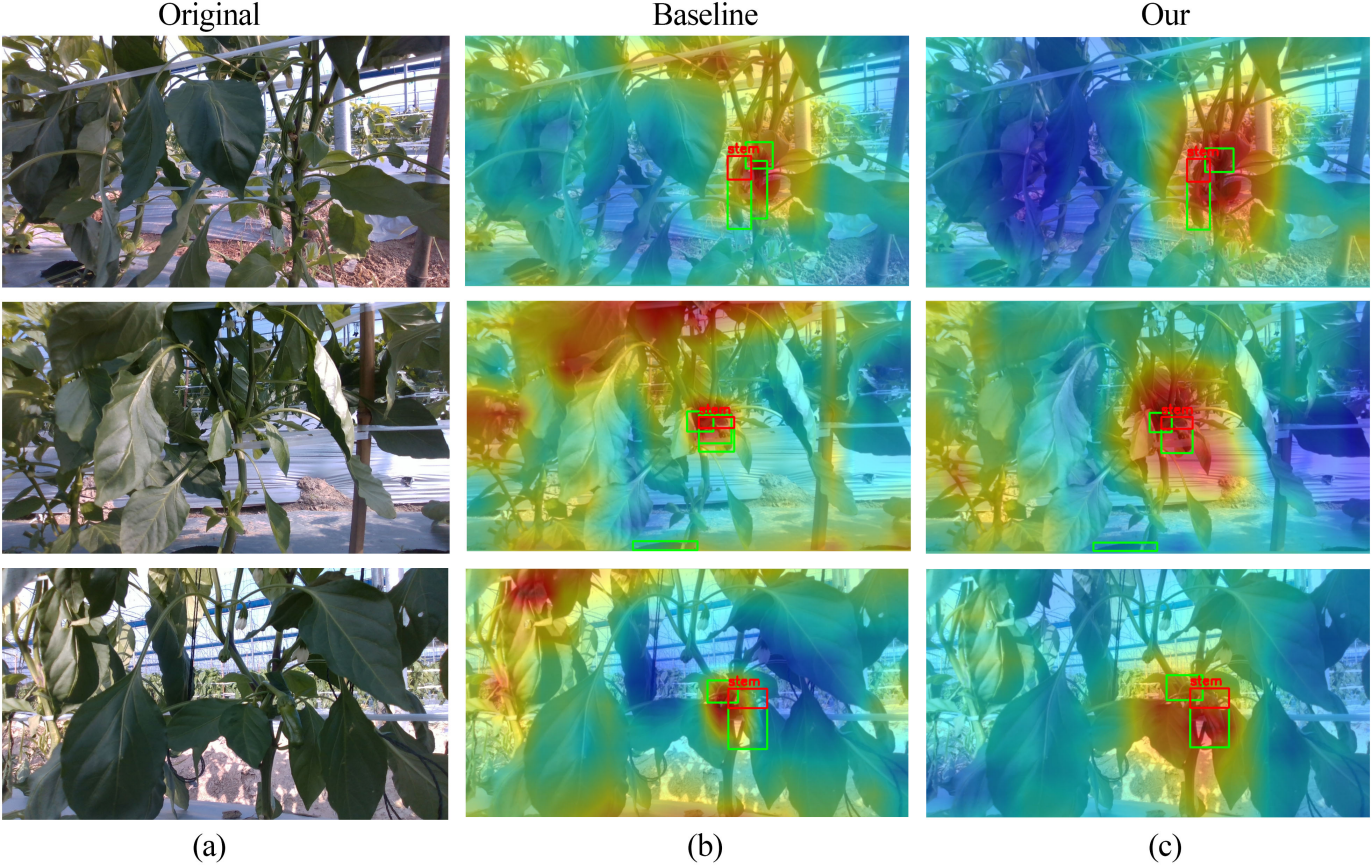
GradCAM++ feature activation comparison between YOLOv11n-pose (baseline) and HAV-Pose on Layer 9 (SPPF). Column **(a)**: original images; column **(b)**: baseline activation maps; column **(c)**: HAV-Pose activation maps. Red boxes indicate the Branch (stem) region; green boxes indicate the DoorPepper region. HAV-Pose exhibits stronger activation within target regions and effective suppression of background interference.

To provide direct visual evidence that HAWConv learns fundamentally different feature representations, **Figure 7** presents GradCAM++ activation maps extracted from Layer 9 (SPPF) for both the baseline and HAV-Pose across three representative scenes exhibiting green-on-green occlusion. Two consistent patterns are observed across all cases. First, the baseline activation maps exhibit dispersed high-response regions extending into semantically irrelevant background areas such as support structures and ground surfaces, indicating that standard convolution fails to suppress background interference under occluded conditions. Second, HAV-Pose produces markedly more concentrated activation centered on the peduncle and pepper regions, with simultaneous suppression of irrelevant background responses. This dual effect of target-region enhancement and background inhibition directly reflects HAWConv’s coupled dual-attention design: the full-rank depthwise channel attention preserves fine-grained inter-channel relationships essential for stem-foliage discrimination, while the subsequent spatial attention operating on the channel-refined feature **F**^′^ further sharpens localization of structurally relevant regions. These qualitative observations are consistent with the quantitative results reported in **Table 4**, collectively confirming that HAWConv captures more discriminative feature representations than CBAM and other baseline attention mechanisms in complex occluded agricultural scenes.

HAWConv demonstrates a favorable trade-off between accuracy and inference efficiency. While modules like CBAM may introduce additional latency due to their multi-step operations, HAWConv benefits from its optimized group convolution design, effectively reducing parameter redundancy. As indicated in **Table 4**, HAWConv achieves a high inference speed of 286 FPS, which outperforms most plug-and-play attention modules (e.g., CA at 256 FPS and SE-Net at 263 FPS). These results confirm that HAWConv is not merely an external plugin but an optimized operator that balances high-precision detection with the real-time constraints of embedded agricultural devices.

### 4.6 Cross-dataset and cross-crop generalization analysis

**Figure 8:**
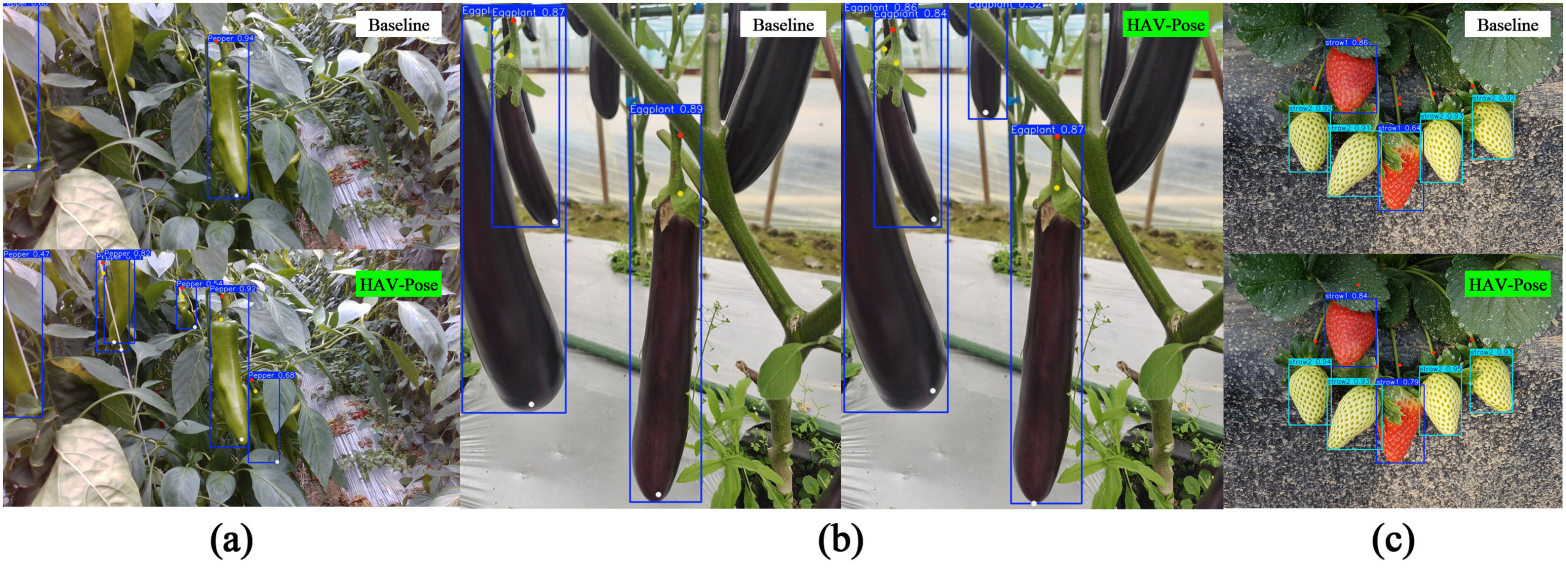
Visual comparison of generalization performance across three public datasets: **(a)** Green Pepper, **(b)** Eggplant, and **(c)** Strawberry. The figure presents qualitative comparisons between the baseline (YOLOv11n-pose) and the proposed HAV-Pose.

To rigorously validate the robustness and cross-domain transferability of HAV-Pose, we extended our evaluation to three publicly available datasets, representing green pepper, eggplant and strawberry, respectively. As summarized in **Table 5**, HAV-Pose demonstrates consistent improvements over the YOLOv11n-pose baseline across all tested domains. To visually corroborate these quantitative gains, **Figure 8** presents a qualitative comparison across the three datasets, highlighting the detection improvements achieved by HAV-Pose over the baseline.

**Table 5:** Generalization performance on three public datasets (Green Pepper, Strawberry, and Eggplant).

Dataset	Model	Object detection (%)				Pose estimation (%)			
		P	R	mAP_50_	mAP_50:95_	P	R	mAP_50_	mAP_50:95_
Green Pepper	YOLOv11n-pose	77.7	76.8	82.8	55.0	79.1	79.5	85.9	80.0
	**HAV-Pose**	83.3	72.7	**85.0**	**58.2**	76.6	80.7	**87.3**	**82.3**
Eggplant	YOLOv11n-pose	88.7	83.5	91.4	67.5	90.6	83.0	92.7	89.8
	**HAV-Pose**	88.4	87.3	**92.9**	**69.7**	88.3	87.0	**93.4**	**90.8**
Strawberry	YOLOv11n-pose	72.3	84.9	85.7	80.7	73.9	71.7	75.4	73.7
	**HAV-Pose**	74.8	86.8	**86.1**	**81.4**	75.8	72.0	**76.3**	**75.5**

Bold values indicate the performance of our proposed HAV-Pose model, which achieves the best results in most metrics.

On the Green Pepper dataset, which shares biological similarities with our primary target (both are *Capsicum* species with slender, elongated peduncles), HAV-Pose achieved object detection and pose estimation mAP_50:95_ scores of 58.2% and 82.3%, respectively, surpassing the baseline by 3.2% and 2.3%. The morphological similarity between green pepper and crown pepper stems facilitates effective feature transfer, suggesting that the texture-aware representations learned by HAWConv transfer across acquisition environments and growth stages within the same botanical family.

On the Eggplant dataset, HAV-Pose achieves the highest performance among the three generalization targets (*Pose*-*mAP*_50_ 93.4%, *Pose*-*mAP*_50:95_ 90.8%), even breaking through the performance saturation exhibited by the baseline (89.8%). This can be attributed to the distinctive morphological characteristics of eggplant: its peduncle is notably thicker and more rigid than pepper stems, presenting a larger and more geometrically stable target for keypoint regression. Furthermore, the strong color contrast between the dark purple fruit body and the green stem provides unambiguous visual boundaries, reducing the “green-ongreen” texture ambiguity that challenges detection in pepper crops. These factors collectively create a more favorable detection condition, allowing HAWConv’s attention mechanism to localize the picking point with minimal interference.

On the Strawberry dataset, HAV-Pose achieves the lowest generalization performance among the three crops (*Pose*-*mAP*_50_ 76.3%), though it still outperforms the baseline (75.4%). This relatively lower performance is primarily attributable to the distinct morphological differences between strawberry and the training domain. Unlike pepper peduncles, strawberry picking points are located on thin, short peduncles that are often partially hidden beneath the fruit body or surrounding leaves, making them structurally dissimilar to the elongated stem targets in the training set. Additionally, the StrawDI dataset was collected in a different geographic region (Huelva, Spain) under distinct illumination conditions, introducing a larger domain gap compared to the other datasets. Despite these challenges, the consistent improvement over the baseline demonstrates that HAWConv’s hybrid attention mechanism retains sufficient discriminative capability to adapt to structurally dissimilar targets.

### 4.7 Quantitative analysis of picking point localization precision

Although mAP offers a global evaluation of detection capabilities, the pixel-level Euclidean distance error of predicted picking points serves as the determinant factor for the success rate of robotic intervention. To evaluate this fine-grained precision, we conducted a quantitative analysis on the test set. Underperforming models (e.g., YOLOv5, YOLOv6, Star-YOLO and the parameter-heavy Mamba-YOLO) were excluded to focus the comparison on competitive candidates.

As presented in **Table 6**, the proposed HAV-Pose demonstrates improved detection capability and consistency. Compared to the YOLOv11 baseline (155 points), HAV-Pose identified the highest number of valid picking points (160 points), highlighting its robustness in complex scenarios. Furthermore, HAV-Pose achieved the lowest standard deviation on the X-axis (3.488 px), outperforming the baseline (4.927 px) and MSPB-YOLO (28.959 px). It is worth noting that while YOLOv12n-pose yielded a marginally lower X-axis mean error (3.9917 px), it failed to detect 12 valid picking points compared to HAV-Pose (148 vs. 160). This suggests that HAV-Pose does not trade recall for precision by selectively processing easier samples; instead, it maintains top-tier localization stability while covering a broader range of difficult targets. This minimized variance indicates that our model provides the most stable coordinate predictions, effectively mitigating the risk of jitter in robotic motion planning.

**Table 6:** Quantitative comparison of pixel-level picking point localization errors on the test set.

Model	Detected Points	X-axis error (px)		Y-axis error (px)		Euclidean error (px)	
		Mean	Std dev	Mean	Std dev	Mean	Std dev
YOLOv8n-pose	149	4.4888	8.1111	9.1792	9.3885	11.3492	11.3814
YOLOv9t-pose	153	5.2215	8.1069	9.8276	10.5667	11.9454	12.5909
YOLOv10n-pose	155	4.7297	4.5927	10.7903	11.4088	12.7655	11.2737
YOLOv11n-pose	155	4.2407	4.9274	10.6181	14.1244	12.1905	14.3492
YOLOv12n-pose	148	**3.9917**	3.5623	11.0793	10.7226	12.4802	10.5164
YOLOv13n-pose	155	4.6394	4.1170	9.9807	10.5698	11.9580	10.3352
Chili-Pepper	142	4.8803	5.7676	10.7421	14.7194	12.5706	15.2025
MSPB-YOLO	143	4.2039	4.5909	**8.8522**	8.9426	**10.6124**	9.1898
YOLO-ALW	145	4.4302	4.5276	9.3270	9.7333	10.9617	10.0844
Pepper-YOLO	141	5.1978	6.2227	9.1544	8.5257	11.4062	9.5982
**HAV-Pose (Ours)**	**160**	**4.1067**	**3.4880**	9.5211	**7.9972**	11.1303	**7.7301**

Bold values indicate the performance of our proposed HAV-Pose model, which achieves the best results in most metrics.

**Figure 9:**
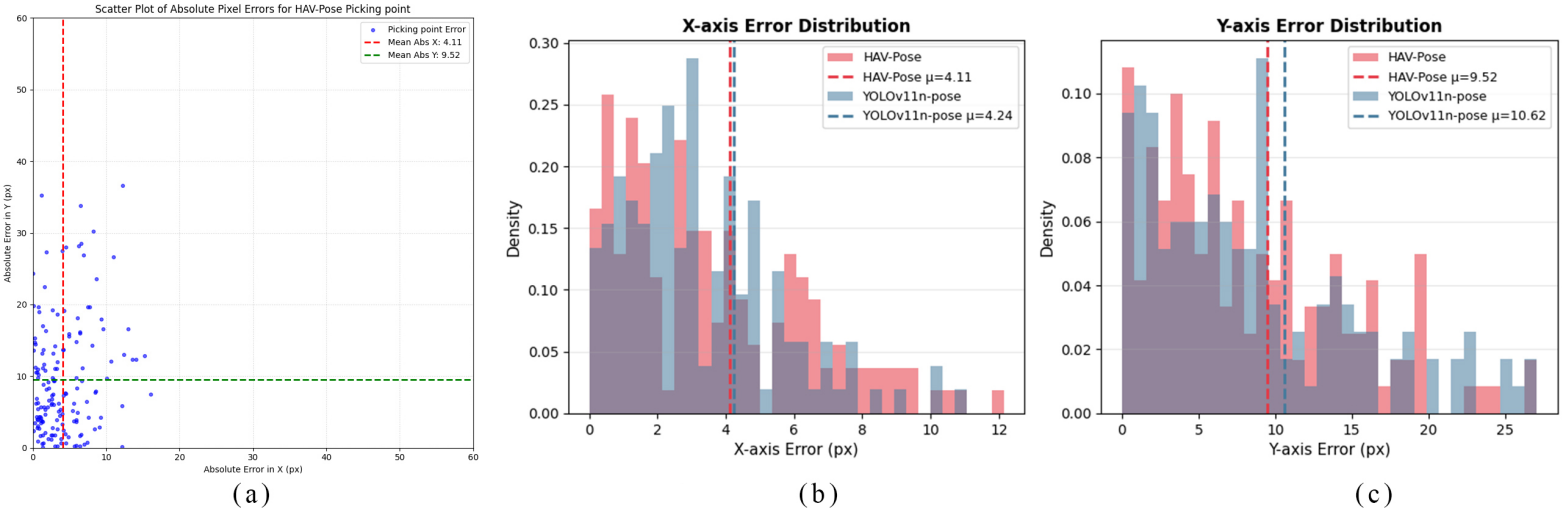
Error analysis of picking point localization for HAV-Pose on the Crown Pepper dataset. **(a)** Scatter plot of absolute pixel errors for HAV-Pose, where dashed lines indicate mean errors in X and Y axes, showing that the majority of predictions are concentrated within low-error regions. **(b)** X-axis error distribution comparison between HAV-Pose and YOLOv11n-pose (baseline), showing that HAV-Pose achieves a higher density in the low-error range with a lower mean error. **(c)** Y-axis error distribution comparison, where HAV-Pose similarly demonstrates a more concentrated distribution around smaller error values.

Crucially, we highlight the concept of Anisotropic Error Tolerance inherent in the harvesting of hanging crops. Given that pepper peduncles are slender, cylindrical structures hanging vertically under gravity, lateral deviation (X-axis error) poses a critical risk of the gripper missing the target entirely. In contrast, longitudinal deviation (Y-axis error) merely results in a shift along the elongated peduncle, typically remaining within the valid picking window. Guided by this operational criterion, **Figure 9** presents a comprehensive error analysis of HAV-Pose on the Crown Pepper test set. As shown in **Figure 9a**, the scatter plot confirms that the vast majority of picking point predictions are concentrated in the low-error region, with mean absolute errors of 4.11px (X-axis) and 9.52px (Y-axis). The error distribution comparisons in **Figures 9b, c** further reveal the advantage of HAV-Pose over the YOLOv11n-pose baseline: on the critical X-axis, HAV-Pose achieves a higher probability density in the 0–3px range and a lower mean error (4.11px vs. 4.24px), indicating superior lateral localization precision. On the Y-axis, HAV-Pose likewise exhibits a more concentrated distribution (9.52px vs. 10.62px). By minimizing critical lateral errors while maintaining the highest detection coverage (160 valid picks vs. 153 for the baseline), HAV-Pose provides more reliable coordinate guidance for autonomous picking systems.

### 4.8 Evaluation of obstacle-aware pose planning

To validate the effectiveness of the proposed Spherical Voxel-based Visibility Field (SVVF) algorithm in unstructured agricultural environments, we conducted comparative experiments focusing on safety margins, robustness, and computational efficiency. The evaluation was performed on a test set stratified by occlusion complexity into three categories: Complex Obstacles (**Figure 1d**), where the target peduncle is surrounded by dense foliage on multiple sides; Stem Side Occlusion (**Figure 1e**), where one lateral side is partially blocked by adjacent stems or fruits; and No Occlusion (**Figure 1f**), where the target peduncle is freely accessible, serving as a reference baseline to quantify planning overhead under unobstructed conditions.

#### 4.8.1 Comparative experimental setup

To rigorously evaluate the performance of the proposed SVVF algorithm, we conducted comparative experiments against two representative baseline strategies commonly employed in robotic harvesting tasks. These baselines serve to verify the necessity of active obstacle avoidance and demonstrate the advantages of the max-margin optimization strategy. Our comparison includes a Fixed-Pose Strategy (Baseline 1) and a Randomized Sampling Strategy (Baseline 2).

Baseline 1: Fixed-Pose Strategy (Deterministic). This method adopts a naive approach where the end-effector approaches the target centroid *P_target_*directly along the camera’s optical axis (or the surface normal), maintaining a fixed orientation. It operates without an active collision avoidance module, serving as a control group to quantify the complexity of the test environments.

Baseline 2: Randomized Sampling Strategy (Stochastic). This method employs a sampling-based logic inspired by RRT (Rapidly-exploring Random Trees) (Cao et al., 2019). It iteratively samples random approach vectors ${\vec{n}}_{rand}$ within the local spherical space and terminates immediately upon identifying the first collision-free path. This represents a “satisficing” strategy that prioritizes feasibility over optimality (i.e., finding a solution rather than the safest one).

#### 4.8.2 Qualitative analysis of decision process

**Figure 10:**
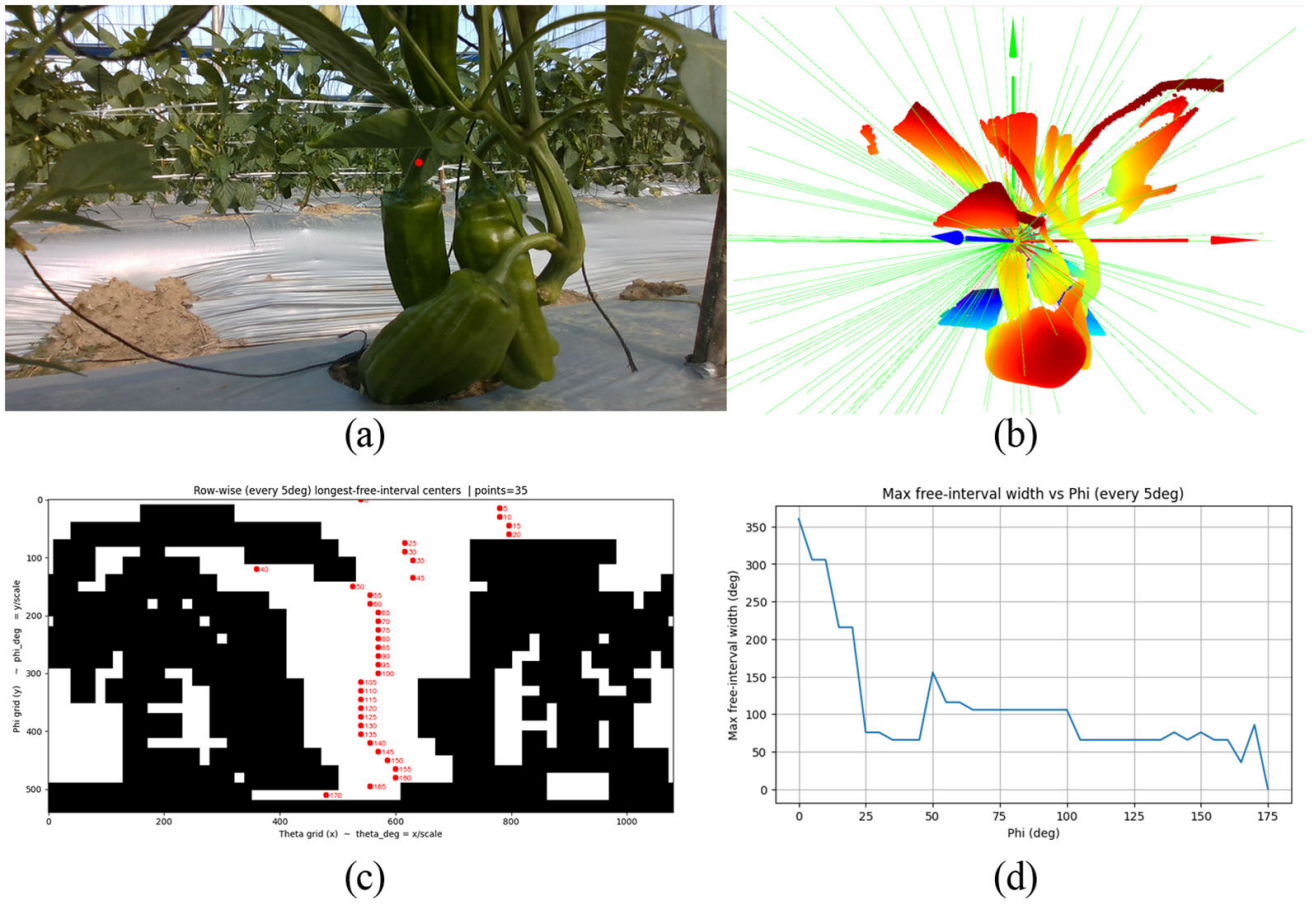
Qualitative visualization of the SVVF perception and analysis pipeline. **(a)** Reference RGB view of the complex harvesting scene with occluding foliage. **(b)** Omnidirectional ray casting converts the depth information into a 3D spatial distribution, where red/yellow rays indicate obstacles and green rays indicate free space. **(c)** The 2D Spherical Visibility Map (SVM). The horizontal and vertical axes correspond to the azimuth *θ* and elevation *ϕ*, respectively. Black regions indicate dilated obstacles, while white regions represent feasible space. The red dots mark the geometric centers of the widest collision-free intervals calculated for each elevation layer, labeled with their corresponding elevation angles (*ϕ*) in degrees. **(d)** The safety score curve quantifies the maximum free-interval width across varying elevation angles (*ϕ*) to identify the optimal insertion plane.

**Figure 11:**
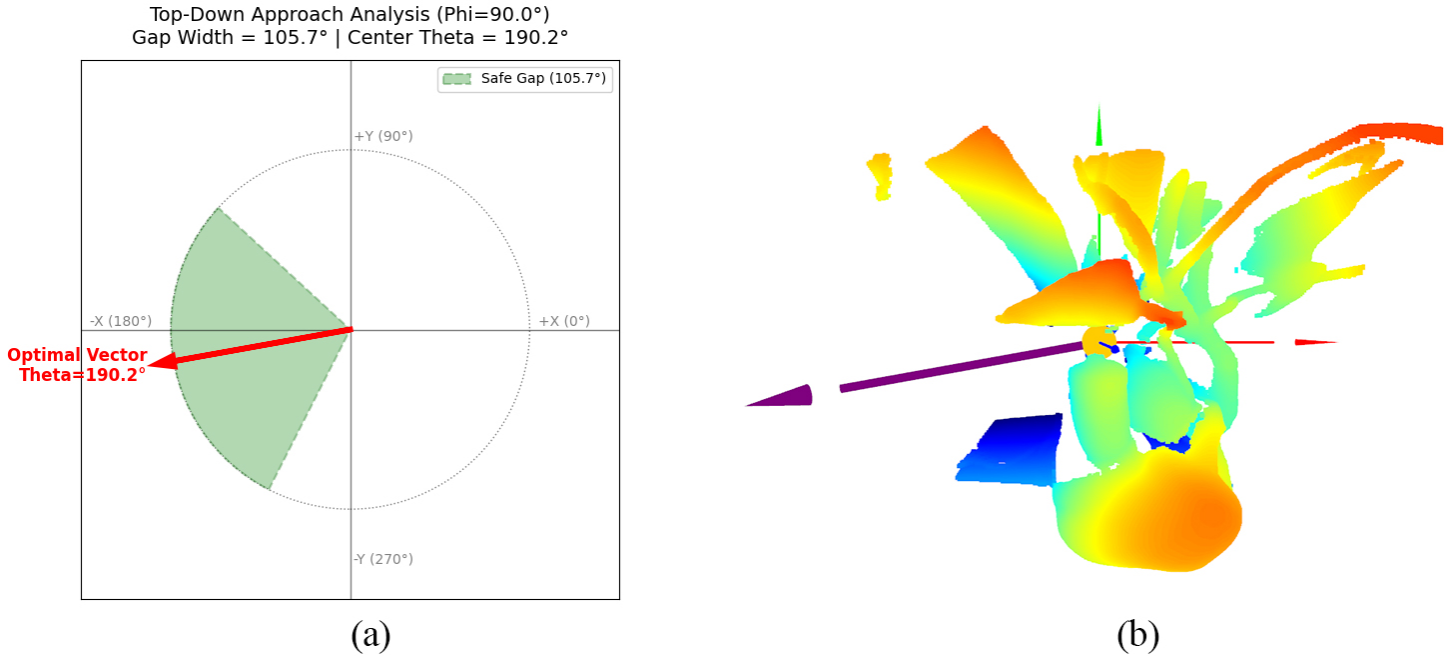
Visualization of the optimal approach decision. **(a)** Top-down polar analysis at the selected elevation plane (*ϕ* = 90^°^). The green shaded sector marks the maximum collision-free gap (width 105.7^°^), and the red arrow (*θ* = 190.2^°^) indicates the optimal approach vector aligned with the gap’s geometric center. **(b)** The corresponding 3D visualization in the point cloud environment. The purple arrow represents the planned end-effector trajectory derived from **(a)**, demonstrating a collision-free insertion path that maximizes clearance from surrounding foliage.

The execution pipeline of the SVVF algorithm is visually reconstructed in **Figures 10**, **11**, demonstrating how the system transforms unstructured environmental constraints into a reliable, mathematically optimal decision.

In the initial perception and mapping phase, the process begins with local scene analysis. As illustrated in the RGB reference view (**Figure 10a**), the target fruit is surrounded by dense foliage and adjacent peppers, posing a high collision risk. To mitigate computational redundancy, the algorithm reconstructs only a local voxelized space centered on the target rather than processing the entire global scene. Within this local region, omnidirectional ray casting is initiated, generating the 3D spatial distribution depicted in **Figure 10b**, where collision-free paths (green rays) and obstacle occlusions (red/yellow regions) are distinctively characterized. To facilitate efficient planning, this volumetric information is projected onto the *θ* − *ϕ* domain to synthesize the Spherical Visibility Map (SVM), as shown in **Figure 10c**. Here, black regions represent physical obstacles expanded by morphological dilation. A row-wise search is then performed along the elevation axis (*ϕ*) to identify the geometric center of the widest continuous free interval for each layer (marked by red dots).

During the subsequent optimization and decision phase, the topological safety is quantified by calculating the maximum gap width for each elevation angle, yielding the safety scoring curve shown in **Figure 10d**. Notably, a high-safety plateau is observed in the middle elevation range. To resolve ambiguity among these equally robust candidates, the algorithm incorporates kinematic preferences, ultimately selecting the horizontal plane (*ϕ* = 90^°^) as the optimal insertion angle. The resulting decision is detailed in **Figure 11**. The polar analysis at the selected plane (**Figure 11a**) reveals a wide safe sector of 105.7^°^. The planner selects the bisector of this sector (*θ* = 190.2^°^, red arrow) to maximize the equidistant safety margin. This theoretical decision is empirically validated in the local point cloud environment shown in **Figure 11b**, where the planned trajectory (purple arrow) successfully navigates through the foliage gap without collision.

#### 4.8.3 Quantitative analysis

The comparative results are summarized in **Table 7**. In the Complex Obstacles scenario, Baseline 1 fails to provide a safe path, exhibiting a 0% robustness rate and a dangerous average clearance of only 4.51 mm. Baseline 2 improves feasibility with a robustness of 56.8%, but its stochastic nature leads to inconsistent safety margins (11.42 mm), often selecting narrow gaps barely wide enough for the gripper.

**Table 7:** Quantitative comparison of planning performance under different obstacle scenarios.

Method	Scenario	Count	Mean min. clearance (mm)	Mean robustness (%)	Mean planning time (ms)
Baseline 1	Complex Obstacles	10	4.51	0	380.00
	Stem Side	27	2.90	2.14	385.11
	No Occlusion	24	2.09	3.75	402.17
Baseline 2	Complex Obstacles	10	11.42	56.80	**346.59**
	Stem Side	27	10.40	39.90	**361.43**
	No Occlusion	24	12.42	52.00	372.20
**SVVF**	Complex Obstacles	10	12.72	**65.40**	413.90
	Stem Side	27	13.58	**65.48**	391.70
	No Occlusion	24	17.33	**76.58**	396.20

Bold values indicate the performance of our proposed HAV-Pose model, which achieves the best results in most metrics.

In contrast, the SVVF method demonstrates improved performance. In the most challenging Complex Obstacles category, it achieves the highest average clearance of 12.72 mm and a robustness score of 65.4%. Notably, in the No Occlusion scenario, the robustness reaches 76.58%, significantly outperforming Baseline 1 (3.75%) and Baseline 2 (52%). This improvement is attributed to the Max-Margin Strategy, which systematically searches for the widest gap to maximize tolerance to control errors.

Regarding efficiency, the proposed method incurs a marginal computational overhead (averaging 413.9 ms in complex scenes) compared to Baseline 2 (346.59 ms). However, considering the mechanical execution time of the arm (typically several seconds), this additional ≈67 ms processing time is negligible. Given the substantial gains in safety reliability (+24.6% robustness over Baseline 2 in No Occlusion), SVVF achieves a more effective trade-off for real-time harvesting.

## 5 Discussion

### 5.1 Synergistic integration of attention-based perception and spatial planning

The “perception-action gap” remains a bottleneck in unstructured agricultural environments. Our results demonstrate that HAV-Pose effectively bridges this gap by integrating high-precision perception with deterministic planning. Our ablation studies and attention mechanism experiments specifically validate the contribution of the original, plug-and-play HAWConv (Hybrid Attention Weighted Convolution) module. Results indicate that removing this channel-level attention significantly degrades detection performance in dense foliage, confirming that HAWConv is not merely a redundant addition but a critical component for suppressing background noise. To ensure that this feature recalibration does not compromise real-time performance, HAWConv is synergized with the RepC3k re-parameterized architecture. This combination allows the visual backend to balance high-dimensional feature extraction with inference speed. Consequently, the physical positioning accuracy experiments reveal that the system maintains millimeter-level precision even under occlusion, ensuring that the perception module provides a reliable centroid (*P_target_*) for the SVVF module without introducing systematic errors.

### 5.2 Comparison of deterministic Max-Margin planning and stochastic sampling

A critical finding from this study is the validation of the Max-Margin Optimization Strategy utilized by the SVVF algorithm over traditional sampling-based methods. Comparative experiments against fixed-pose and RRT-based baselines highlight the limitations of stochastic methods in constrained spaces. As evidenced in the quantitative analysis (Section 4.8.3), Baseline 2 (RRT-inspired strategy) exhibited a high variance in safety clearance. This is inherent to the “satisficing” nature of stochastic algorithms, which terminate upon finding any feasible path, often selecting narrow gaps that barely accommodate the gripper (Zhang et al., 2026).

This limitation is visually corroborated by the qualitative analysis in **Figure 10a**, where the target Crown Pepper is physically sandwiched between a rigid main stem and dense foliage. In such “green-on-green” scenarios, a naive fixed-pose attempt (Baseline 1) inevitably leads to collision (0% success rate). Similarly, while a stochastic planner might identify a feasible yet narrow path near the stem, it imposes a high risk of damage under mechanical vibration. These physical constraints underscore the necessity for a planner that does not merely find a path, but explicitly maximizes safety.

In contrast, the SVVF algorithm transforms the 3D unstructured environment into a 2D Spherical Visibility Map (SVM), essentially performing an exhaustive search in a reduced dimensional space. This allows the planner to identify the global maximum safety interval (e.g., the geometric center of the widest 105.7^°^ sector). The substantial lead in robustness rate (65.4% vs. 56.8%) observed in our comparative analysis suggests that this deterministic behavior offers an advantage over random sampling, particularly in identifying mechanically stable approach vectors (*ϕ* = 90^°^). This ensures consistent and predictable operation in varying crop densities.

### 5.3 Robustness and generalization across varied environments

Beyond detection accuracy, HAV-Pose exhibits consistent generalization capability across diverse growing conditions, as validated by cross-dataset experiments spanning four distinct datasets with varying lighting conditions, crop varieties, and planting densities. The consistent performance achieved without retraining suggests that the feature representations learned by HAWConv are robust to domain shifts, addressing a critical bottleneck in deep learning-based agricultural robotics. The robustness gains observed in complex scenarios further indicate that explicitly modeling the obstacle space is a more effective strategy than relying on stochastic sampling approaches.

**Figure 12:**
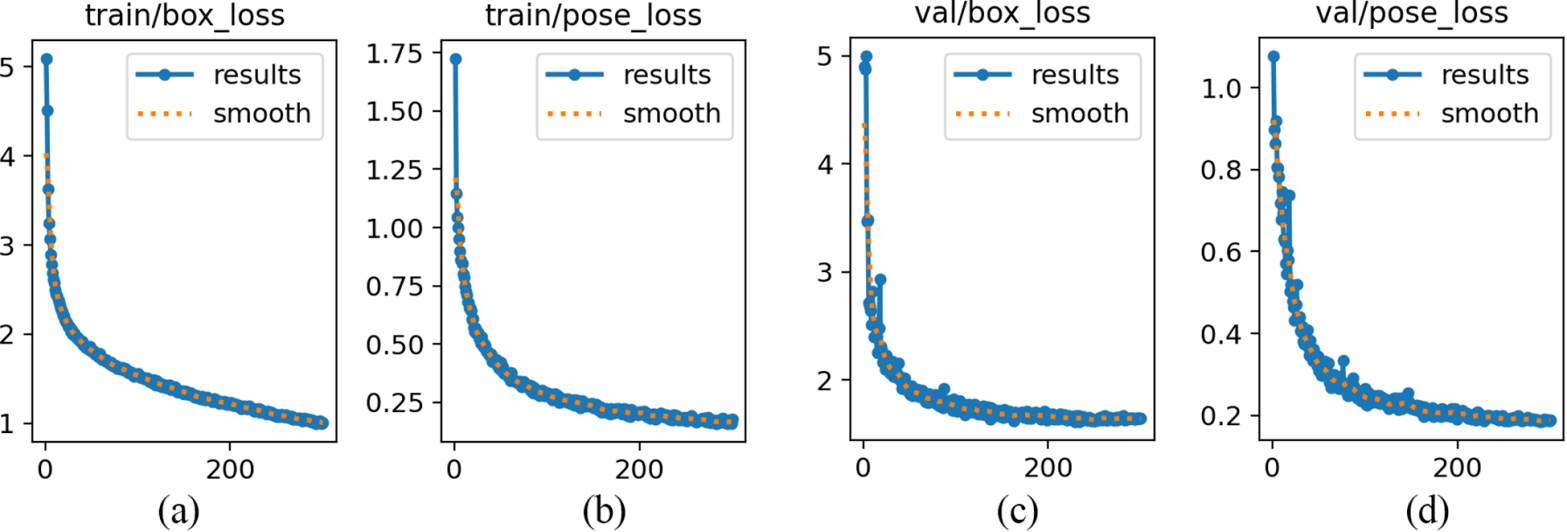
Training and validation loss curves of HAV-Pose over 300 epochs on the Crown Pepper dataset. **(a)** Training box loss; **(b)** training pose loss; **(c)** validation box loss; **(d)** validation pose loss. All four curves decrease smoothly and consistently throughout training, with the validation loss trajectories closely tracking their training counterparts without observable upward divergence, confirming stable convergence and the absence of significant overfitting.

The reliability of these generalization results is further substantiated by the stability of the training process itself. As shown in **Figure 12**, the box loss and pose loss for both training and validation sets decrease smoothly and consistently across all 300 epochs, with the validation loss curves closely tracking their training counterparts without any observable upward divergence. The absence of a generalization gap between training and validation trajectories confirms that HAV-Pose does not overfit to the relatively small Crown Pepper training set (1,246 images, 3,185 annotated instances), but instead learns robust and transferable feature representations. This training stability, combined with the consistent cross-dataset performance across four tructurally distinct crop datasets, collectively validates that the performance gains reported in this study reflect genuine improvements in feature discrimination capability rather than dataset-specific memorization.

### 5.4 Limitations and future work

Despite the promising results, this study has limitations. First, the current SVVF relies heavily on the quality of the depth map; in scenarios with extreme lighting conditions (e.g., direct sunlight causing depth dropouts) or thin obstacles (e.g., wires) that are below the depth sensor’s resolution, the voxel reconstruction may be incomplete. Second, the current planning is static; it assumes the environment is rigid during the computation. Future work will focus on two directions: (1) integrating RGB-D fusion techniques to enhance obstacle geometric reconstruction in varying lighting conditions, and (2) extending SVVF to dynamic obstacle avoidance, allowing the manipulator to react to moving branches or wind disturbances in real-time.

## 6 Conclusion

In this study, we proposed HAV-Pose, an integrated obstacle-aware pose estimation framework designed to bridge the “perception-action gap” in robotic harvesting. To ensure high-precision perception in unstructured environments, the system incorporates the original HAWConv module synergized with the RepC3k re-parameterized architecture. This combination leverages HAWConv for attention-driven feature refinement to suppress background foliage noise, while utilizing RepC3k to ensure efficient real-time inference. Bridging perception and execution, the SVVF algorithm was developed to transform 3D obstacles into a 2D Spherical Visibility Map. By employing a Max-Margin Optimization Strategy, the planner deterministically identifies the global optimum—the geometric center of the widest collision-free interval.

Extensive evaluations were conducted to validate the system’s efficacy. Ablation studies confirmed the essential contribution of the HAWConv attention mechanism, while cross-dataset validation across four diverse datasets demonstrated the model’s generalization capabilities. Notably, in the most challenging complex occlusion scenarios, HAV-Pose achieves a planning robustness rate of 65.4%. This represents an improvement over the stochastic baseline (56.8%) and a notable improvement compared to the failure of the fixed-pose strategy (0%). Furthermore, with a marginal computational overhead (≈ 67 ms), the system achieves a favorable trade-off between safety and efficiency. These results confirm HAV-Pose as a viable solution for commercial agricultural robots. Future work will focus on extending the framework to dynamic environments to adapt to moving obstacles and integrating active perception strategies to further mitigate occlusion challenges.

## Data Availability

Data will be made available on request.
